# Supplementary material for: Optimization of ultrasonic-assisted extraction of phenolic compounds from Clinacanthus nutans using ionic liquid (ILs) binary solvent: Application of Peleg’s model and response surface methodology
Source: PLoS One. 2025 Jul 3;20(7):e0326141. doi: 10.1371/journal.pone.0326141 (PMC12225847; doi:10.1371/journal.pone.0326141)
Supplement: Attachment — Submitted filename: Corrections List 18 April 2025.docx [file pone.0326141.s003.docx]

Dear Editor-in-Chief,

Attached herewith are the lists of second corrections for your kind perusal. Comments from reviewers and responses to the comments for the manuscript ‘**Optimization of Ultrasonic-Assisted Extraction of Phenolic Compounds from *Clinacanthus nutans* using Ionic Liquid (ILs) Binary Solvent: Application of Peleg’s Model and Response Surface Methodology**’ are as follows:

**Reviewer 1:**

| **No** | **Reviewer’s Comments and Questions** | **Corrections and Response** |
| --- | --- | --- |
| **1.** | **Introduction**  The present study describes the optimization of an extraction method for the recovery phenolic compounds from *Clinacanthus nutans* plant using DES as extractor medium. Although the authors present several pharmacological properties in introduction, they did not correlate them with specific phenolic compounds. Thus, they performed simple photometric assays as TPC, DPPH and FRAP to carry out the optimization. Are there any correlation of these measurements with specific biological or pharmacological activity? Although the extraction parameters are optimized, the usefulness of extraction is uncertain. | Authors have added the pharmacological activity of the phenolic compounds as such in the manuscript. Phenolic compounds, characterized by one or more hydroxyl groups attached to an aromatic hydrocarbon chain, display diverse biological activities primarily due to their antioxidant properties. Research indicates that TPC is often positively correlated with antioxidant activities across different plant extracts. For instance, studies have demonstrated that higher concentrations of phenolic compounds correspond to increased radical scavenging activity as exhibited in DPPH assays, suggesting that phenolic compounds play substantial roles in providing antioxidant effects due to their ability to donate electrons (Valdez‐Morales et al., 2014). Notably, it has been shown that specific phenolic acids exhibit inhibitory effects on tyrosinase (a key enzyme in melanin synthesis) by forming hydrogen bonds with the enzyme, further emphasizing the functional relevance of phenolic structures (Kim et al., 2021). certain phenolic compounds are linked to broader pharmacological activities such as anti-inflammatory and antimicrobial properties (Albuquerque et al., 2021). |
| **2.** | **Specific comments**  1. Please explain why did you use this specific DES? A wide range of DES solvents are available, Did you perform prelimenary experiments? | Authors selected choline chloride and 1,4-butanediol as the DES based on prior studies demonstrating their effectiveness in phenolic extraction and favorable green chemistry properties. Although authors did not conduct a full comparative screening of other DES types, this combination was chosen for its moderate polarity, low toxicity, and suitability for ultrasound-assisted extraction. Preliminary optimization using different IL-to-water ratios (2:8, 5:5, 8:2) confirmed its effectiveness, as supported by our RSM and Peleg model results. Future work will include comparisons with other DES systems to further validate and enhance extraction efficiency.  Othman, Z. S., Hassan, N. H., & Zubairi, S. I. (2017). Response surface optimization of rotenone using natural alcohol-based deep eutectic solvent as additive in the extraction medium cocktail. Journal of Chemistry, 2017, 1-10. <https://doi.org/10.1155/2017/9434168>  Cao, J., Yang, M., Cao, F., Wang, J., & Su, E. (2017). Well-designed hydrophobic deep eutectic solvents as green and efficient media for the extraction of artemisinin from artemisia annua leaves. ACS Sustainable Chemistry &Amp; Engineering, 5(4), 3270-3278. https://doi.org/10.1021/acssuschemeng.6b03092 |
|  | 2. Which is the usefulness of Peleg’s model? | Authors have added the information on the usefulness of Peleg’s model in the manuscript. |
|  | 3. Why did you use an other plant material as benchmark? | Authors have added information on the reason of using *Strobilanthes crispus* as benchmark. |

**Reviewer 2**

| **No** | **Reviewer’s Comments and Questions** | **Corrections and Response** |
| --- | --- | --- |
|  | **Abstract**  Line 29: The reported TPC value of "0.01 ± 7.97 × 10⁻⁵ mg GAE/g" appears inconsistent with the earlier reported value of "42.556 ± 0.0003 mg GAE/g" (Line 25). This contradiction needs clarification to avoid reader confusion. | - Authors acknowledge the inconsistency and confirm that it resulted from a typographical error in the manuscript. - The value of 42.556 ± 0.0003 mg GAE/g refers to the experimental TPC obtained at 50 kHz, as determined during Peleg’s model analysis (this is the earlier phase of the OFAT experimental). - However, the predicted TPC value from the RSM optimization was 0.01 ± 7.97 × 10⁻⁵ mg GAE/g, which corresponds to the optimal extraction conditions. - This statement was added in the conclusion part for clarification: ….”However, the discrepancy between the TPC values obtained from the Peleg model and RSM optimization is primarily due to the differing objectives and experimental conditions of each approach in which Peleg's model reflects specific experimental data at 50 kHz, while RSM predicts outcomes based on optimized variable combinations. Additionally, the RSM-predicted value may be influenced by model extrapolation or scaling limitations, leading to an unrealistically low estimate….” |
|  | **Introduction**   - Line 45: The section provides a comprehensive ethnopharmacological background but lacks a clear identification of the research gap. - Line 91: Consider explicitly stating the novelty of applying IL binary solvents with Peleg’s model and RSM in combination, as this is the unique contribution of the study. Also, justification why Peleg’s model was chosen for deciding the optimum extraction time instead of combining the independent variable of time in the CCRD experimental design. - Please clearly justify the needed of using Peleg’s model over other kinetic models. How does it offer superior insight for this type of extraction? | - The suggestions on the clear identification of the research gap has been added in the introduction section. - The suggestions on the novelty of applying IL binary solvents with Peleg’s model and RSM in combination and justification why Peleg’s model was chosen for deciding the optimum extraction has been added been added in the introduction section. - The justification on the need of using Peleg’s model over other kinetic models has been added in the introduction section. |
|  | **Methodology**   - Line 94: The addition of Strobilanthes crispus as a benchmark is interesting but underexplained. The rationale for its inclusion should be made explicit. - Line 117: The drying process may affect compound stability. Please clarify how compound degradation due to heat was prevented or controlled. - Line 180: No information is provided about the temperature control during UAE. This is critical because ultrasound generates heat which can degrade phenolic compounds. This should be addressed. - Line 254: While ANOVA is used, consider reporting effect sizes or regression coefficients to provide deeper insights into variable impact. | - Authors have added information on the reason of using *Strobilanthes crispus* as benchmark in the introduction section. - Authors have added information on how compound degradation due to heat was prevented in the methodology section. - The information about the temperature control during UAE have been added into section 2.7. - Thank you for this thoughtful suggestion. In our analysis, we primarily relied on the regression coefficients generated through the RSM model to reflect the magnitude and direction of each variable’s effect on the responses. These coefficients are presented in Table 5 and form the basis of our 3D surface plots and optimization analysis. While standardized effect size metrics such as η² were not reported separately, we believe the regression equations, along with the ANOVA results and surface plots, sufficiently capture the influence and interaction of the independent variables. We have clarified this interpretation in the revised manuscript to improve transparency and provide more intuitive insights into variable impact. |
|  | **Results and Discussion**   - Line 306: The R² values are low (especially for DPPH: 0.048), which significantly undermines model predictability. The manuscript should discuss limitations of the model and perhaps consider model refinement or alternate modeling approaches. - Line 408: There is conflicting interpretation regarding whether lower or higher ultrasonic frequencies enhance FRAP. Authors should clarify or reanalyze the statements to maintain consistency. - Line 492: The comparison between UAE and traditional extraction methods is important, but should discuss the potential trade-off between yield and compound preservation due to ultrasound-induced degradation. - While the use of ILs and Peleg’s model is appropriate, deeper discussion of solvent interaction mechanisms (e.g., hydrogen bonding, viscosity effects) should be integrated to enrich the explanation beyond statistical outcomes. | - Authors acknowledge that the R² value for the DPPH model is low (0.048), indicating limited predictive strength for this particular response variable under the applied quadratic model. This likely reflects the complex behavior of antioxidant activity influenced by multiple interacting factors not fully captured by the current two-variable design. In response, authors have added a discussion of this limitation in the manuscript, noting that while the model is statistically valid, its predictive capacity for DPPH is weak. Authors have also clarified that, despite this, the model was useful in identifying general trends and guiding optimization. For future studies, authors agree that incorporating additional independent variables (e.g., temperature or extraction time) or exploring non-linear or machine learning-based models (e.g., artificial neural networks or support vector regression) may improve model fit and predictive performance. - Authors have revised the text to reflect a consistent interpretation, while both lower and higher frequencies can enhance extraction through different mechanisms, in our study, higher ultrasonic frequencies (60 kHz and above) were associated with greater FRAP values, likely due to increased cavitation intensity and enhanced mass transfer. Authors have reworded the relevant section to ensure interpretive consistency and clearer explanation of the observed trend. - Authors agree that while UAE improves extraction yield and efficiency, it may also pose a risk of compound degradation due to localized heat and free radical formation from cavitation. Authors have revised the comparison section to reflect this trade-off, acknowledging that although UAE enhances extraction kinetics, care must be taken to control processing conditions to preserve thermolabile bioactives. - Authors have expanded the discussion to include relevant considerations such as hydrogen bonding, viscosity, and polarity effects associated with the IL–water mixtures used in this study. |
|  | **Conclusion**   - Line 537: The suggestion for future research is valid but broad. It could be strengthened by proposing a more specific HPLC analysis plan or mentioning which phenolic compounds are suspected to be dominant. - Line 541: Temperature control during ultrasonic extraction is listed as a future consideration—this should also be discussed in the Methods or Results section. | - Authors have a new section to propose a more specific HPLC analysis plan as suggested by the reviewer. - The information about the temperature control during UAE have been added into section 2.7. |
|  | **Overall**   - Keywords (Line 42): Consider revising keywords to be more distinct from the title to enhance discoverability (e.g., “green solvent extraction,” “Clinacanthus nutans bioactives”). - Nomenclature: The scientific name Clinacanthus nutans should be italicized throughout the text. - Figures and Tables: Ensure all figures are referenced in the text in sequential order. Currently, some figure explanations (e.g., Figure 10) appear without context. | - Authors have revised the keywords to reduce overlap with the title as suggested by the reviewer. - Authors have carefully reviewed the manuscript and ensured that the scientific name *Clinacanthus nutans* is consistently italicized throughout the text as suggested by the reviewer. - Authors have reviewed the manuscript and ensured that all figures are referenced in the main text in proper sequential order. Authors have added explanation for all figures as suggested by the reviewer. |
|  | **Reference**   - Alphabetize the reference list and ensure uniformity in journal naming (italicization, abbreviations). - Some citations are quite dated (e.g., 2000–2009). Consider adding more recent references (within the last 5 years) to support recent claims about DES and ultrasonic-assisted extraction mechanisms | - The reference list has been thoroughly reviewed and revised to ensure all entries are now alphabetized correctly. Additionally, journal titles have been standardized for consistency using uniform abbreviations and italicization following the journal’s required referencing style. - Authors have added recent references to the manuscript as suggested by the reviewer. |

Best regards,

**Assoc. Prof. Ts. Dr. SAIFUL IRWAN ZUBAIRI**

Ph.D (Chemical Engineering) Imperial College London, M.Eng. (Bioprocess), B.Eng. (Hons.)(Chemical-Bioprocess) UTMalaysia | P.Tech. (MBOT), AMIChemE, PMIFT, Grad. (BEM)

Department of Food Sciences, Faculty of Science & Technology,

Universiti Kebangsaan Malaysia, 43600 UKM Bangi, Selangor.

+603-8921-5989 | [saiful.zubairi08@alumni.imperial.ac.uk](mailto:saiful.zubairi08@alumni.imperial.ac.uk) | [saiful-z@ukm.edu.my](mailto:saiful-z@ukm.edu.my)
